# Connecting post-release mortality to the physiological stress response of large coastal sharks in a commercial longline fishery

**DOI:** 10.1371/journal.pone.0255673

**Published:** 2021-09-15

**Authors:** Nicholas M. Whitney, Karissa O. Lear, John J. Morris, Robert E. Hueter, John K. Carlson, Heather M. Marshall

**Affiliations:** 1 Anderson Cabot Center for Ocean Life, New England Aquarium, Boston, Massachusetts, United States of America; 2 Centre for Sustainable Aquatic Ecosystems, Harry Butler Institute, Murdoch University, Murdoch, Western Australia, Australia; 3 Center for Shark Research, Mote Marine Laboratory, Sarasota, Florida, United States of America; 4 OCEARCH, Park City, Utah, United States of America; 5 Southeast Fisheries Science Center, National Oceanic and Atmospheric Administration, Panama City, Florida, United States of America; 6 State College of Florida, Bradenton, Florida, United States of America; Institut de Recherche pour le Developpement, FRANCE

## Abstract

Bycatch mortality is a major factor contributing to shark population declines. Post-release mortality (PRM) is particularly difficult to quantify, limiting the accuracy of stock assessments. We paired blood-stress physiology with animal-borne accelerometers to quantify PRM rates of sharks caught in a commercial bottom longline fishery. Blood was sampled from the same individuals that were tagged, providing direct correlation between stress physiology and animal fate for sandbar (*Carcharhinus plumbeus*, N = 130), blacktip (*C*. *limbatus*, N = 105), tiger (*Galeocerdo cuvier*, N = 52), spinner (*C*. *brevipinna*, N = 14), and bull sharks (*C*. *leucas*, N = 14). PRM rates ranged from 2% and 3% PRM in tiger and sandbar sharks to 42% and 71% PRM in blacktip and spinner sharks, respectively. Decision trees based on blood values predicted mortality with >67% accuracy in blacktip and spinner sharks, and >99% accuracy in sandbar sharks. Ninety percent of PRM occurred within 5 h after release and 59% within 2 h. Blood physiology indicated that PRM was primarily associated with acidosis and increases in plasma potassium levels. Total fishing mortality reached 62% for blacktip and 89% for spinner sharks, which may be under-estimates given that some soak times were shortened to focus on PRM. Our findings suggest that no-take regulations may be beneficial for sandbar, tiger, and bull sharks, but less effective for more susceptible species such as blacktip and spinner sharks.

## Introduction

Sharks are considered more vulnerable to fishing pressure than most teleosts due to their slow growth, late maturity, and low fecundity [1–3]. In recent years, this concern has led to management initiatives aimed at reducing shark bycatch mortality (i.e., non-targeted incidental capture mortality), prohibiting commercial landing of vulnerable species, and encouraging no-take fishing in recreational fisheries [4,5]. While these methods undoubtedly reduce total fishing mortality, bycatch mortality remains one of the leading factors contributing to shark population declines worldwide [6,7]. Bycatch mortality can be considered in two categories: at-vessel mortality, and post-release mortality. At-vessel mortality (AVM), wherein animals are already dead upon capture, can be quantified and reported relatively easily and subsequently accounted for in fisheries assessments and management. Post-release mortality (PRM) takes place after the animals are released and is caused by physical trauma and physiological effects of capture stress [8–10]. Rates of PRM vary widely between species, gear type, handling practices, and location [11], and thus are difficult to quantify. As a result, fisheries modelers and managers are often forced to estimate PRM rates using data from other species, other fisheries, or calculations based on the AVM rate for a species. This can be problematic since estimates of PRM that are not derived empirically from the species and fishery in question can lead to gross underestimation of fisheries impacts on shark populations [12–14]. Additionally, the implementation of no-take regulations is likely to increase the number of live discards, making it even more essential to quantify PRM rates in order to accurately measure the efficacy of management regulations and the impact of a fishery on a given stock.

Despite the importance of understanding PRM, this information is available for very few shark species and fisheries, as tracking animal fate after release is time-intensive and costly. Most recent shark PRM studies have used either acoustic [e.g. 15–17] or satellite archival tags (PSATs; [e.g. 18–25]) to assess mortality. These tags can infer post-release fate (survived or died) based on depth or tag reporting patterns, but often require maintenance of costly receiver arrays or cost thousands of dollars (USD) for each single-use tag, even in studies using newer, more cost-effective survivorship pop-up archival tags [e.g. 26–28]. These challenges make it difficult to obtain the large sample sizes recommended for calculating high-confidence PRM estimates [e.g. 29–31].

A more economical approach to studying PRM is to focus on at-vessel metrics, including blood stress physiology, which may be useful in predicting animal fate when considered with other metrics [32]. The high-intensity, exhaustive swimming exhibited by hooked sharks causes metabolic and respiratory acidosis in the myotomal tissues, producing cell damage that can impact behavior and cause PRM [reviewed by 8,9,33]. These biochemical changes will be reflected in a blood sample, which can be obtained at the time of capture, and used to understand potential physiological drivers of mortality. As more interspecific data are collected, understanding the relationship between blood stress physiology and mortality may allow a shark’s post-release fate to be predicted based on a simple blood sample in scenarios where tagging may not be possible. However, although the effects of capture on blood physiology have been studied in various shark species [e.g. 9,34–42], most blood studies have not linked their results directly to animal mortality. Only a handful of studies have directly compared blood parameters with post-release fate in sharks [9,16,17,26–28,38,43,44], and these have often investigated recreational fisheries with short fight durations, had relatively small sample sizes, and recorded few mortalities. Overall, blood stress values remain disconnected to empirical post-release fate, and this has limited the value of at-vessel blood stress metrics for estimating mortality.

Our study represents a large-scale effort to link physiology and other at-vessel metrics with actual post-release fate for sharks in a commercial bottom longline (BLL) fishery. We collected blood samples from the same individuals we tagged with acceleration data loggers (ADLs) to assess post-release mortality. Fine-scale depth and swimming data from ADLs provides unambiguous mortality and recovery information [45] and the tags can be reused, allowing increased sample sizes without increasing tag costs [46]. Fishing in the Gulf of Mexico and Florida Keys, we targeted several of the most commonly caught species in the large coastal shark BLL fishery in the U.S. Atlantic region, with a focus on sandbar (*Carcharhinus plumbeus*) and blacktip (*C*. *limbatus*) sharks as well as tiger (*Galeocerdo cuvier*), bull (*C*. *leucas*), and spinner (*C*. *brevipinna*) sharks. Sandbar sharks were the most commonly caught species in this fishery [47], and were assessed as overfished, with overfishing occurring, in 2008 [48]. Their take has been prohibited in the commercial fishery except for a small amount of quota by a limited number of fishers (5–10) under 100% observer coverage [48]. Despite being prohibited and a careful monitoring of the quota, assessments have shown that they are still overfished, and that commercial discards average ~78 metric tons [49]. Blacktip sharks are the second most commonly caught species in this fishery [50] and, based on their blood stress values, are thought to be more susceptible to post-release mortality than other species [37,39,40]. Several recent studies have examined their PRM in recreational fisheries [17,27,44,45,51] but none have done so in a BLL fishery. Tiger, bull, and spinner sharks have not been the subject of recent population assessments in the region but, together with sandbar and blacktip, make up five of the seven most commonly caught sharks in the large coastal shark fishery in the Atlantic region [50]. The PRM rates of these species have also not been previously reported in BLL fisheries. To our knowledge, this study represents the first direct linkage of at-vessel indicators and empirically derived post-release fate for large numbers of these shark species in a BLL fishery.

## Material and methods

### Shark capture and tagging

Experimental bottom longline sets were conducted on contracted commercial bottom longline fishing vessels. Sharks were caught and released near Madeira Beach, FL, and Key West, FL, USA under state permit #SAL-12-0041-SRP issued by the Florida Fish and Wildlife Conservation Commission, and permit #SHK-EFP-1310 issued by the National Marine Fisheries Service Highly Migratory Species Management Division. All procedures were reviewed by the Mote Marine Laboratory Institutional Animal Care and Use Committee and approved under protocol #13-11-NW2. Sharks were caught on standard bottom longline gear consisting of 4.0mm 1200# test monofilament mainline with 3m long, 3.5mm 900# test monofilament gangions terminating with a 18/0 circle hook in sets of up to 260 hooks on 3–6 NM of mainline. Soak times (time from the first hook in the water to the last hook out of the water) ranged from 2–18 h. Hook timers (model HT-600, Lindgren-Pitman, Inc, Pompano Beach, FL) were deployed with each gangion so the actual time on the line (TOL) for each animal was recorded. Specific fishing locations and gear specifications were directed by commercial longline captains to ensure consistency with industry practices. Oceanographic conditions including water temperature, salinity, and dissolved oxygen levels were measured in the middle of the water column for each set using a hand-held meter (YSI model Pro Plus, Yellow Springs, OH, USA).

Hooked sharks were controlled by monofilament leaders and briefly held on deck for tagging and blood sampling. Since this handling protocol was longer than typical fishing practices (in which sharks are often released boat-side), we mitigated these differences by irrigating the gills with seawater and closely monitoring handling time to test as a factor affecting PRM or recovery time. As soon as the shark was secured onboard, a blood sample was taken via caudal venipuncture with a heparinized (Lithium heparin #374858, Sigma-Aldrich, St. Louis, MO, USA) 18 gauge needle. Sex, girth, and total length (TL) of each shark were recorded. Hook location and any visible abrasions, bleeding, or other injuries were noted. An ADL float package (see below) was attached to sharks that were alive and large enough (> ~100 cm TL) to tag. Sharks that were dead at-vessel or that were too small to carry a tag were also measured and their blood was sampled when possible.

Prior to release, hooks were quickly removed or the leaders cut, depending on hook depth, and the individual’s reflexes (nictitating membrane, flex, bite) were tested [52]. The nictitating membrane reflex was tested by squirting 1 ml seawater at the eye from a syringe and noting membrane movement. Bite reflex was assessed by whether the shark would bite the irrigation hose when moved in its mouth, and body flex was assessed as present if the shark flexed its body to either side as it was released. All reflexes were categorized as a 1 if the reflex was unimpaired, or a 0 if the reflex was impaired or absent [52]. Upon release, equilibrium reflex was assessed as a 1 if the shark was able to maintain equilibrium (dorsal fin up) and a 0 if it was not. Each shark was also assigned a condition index score ranging from 1–5 based on their swimming strength and behavior [53], as described in S1 Table.

### Accelerometry

**Fig 1:**
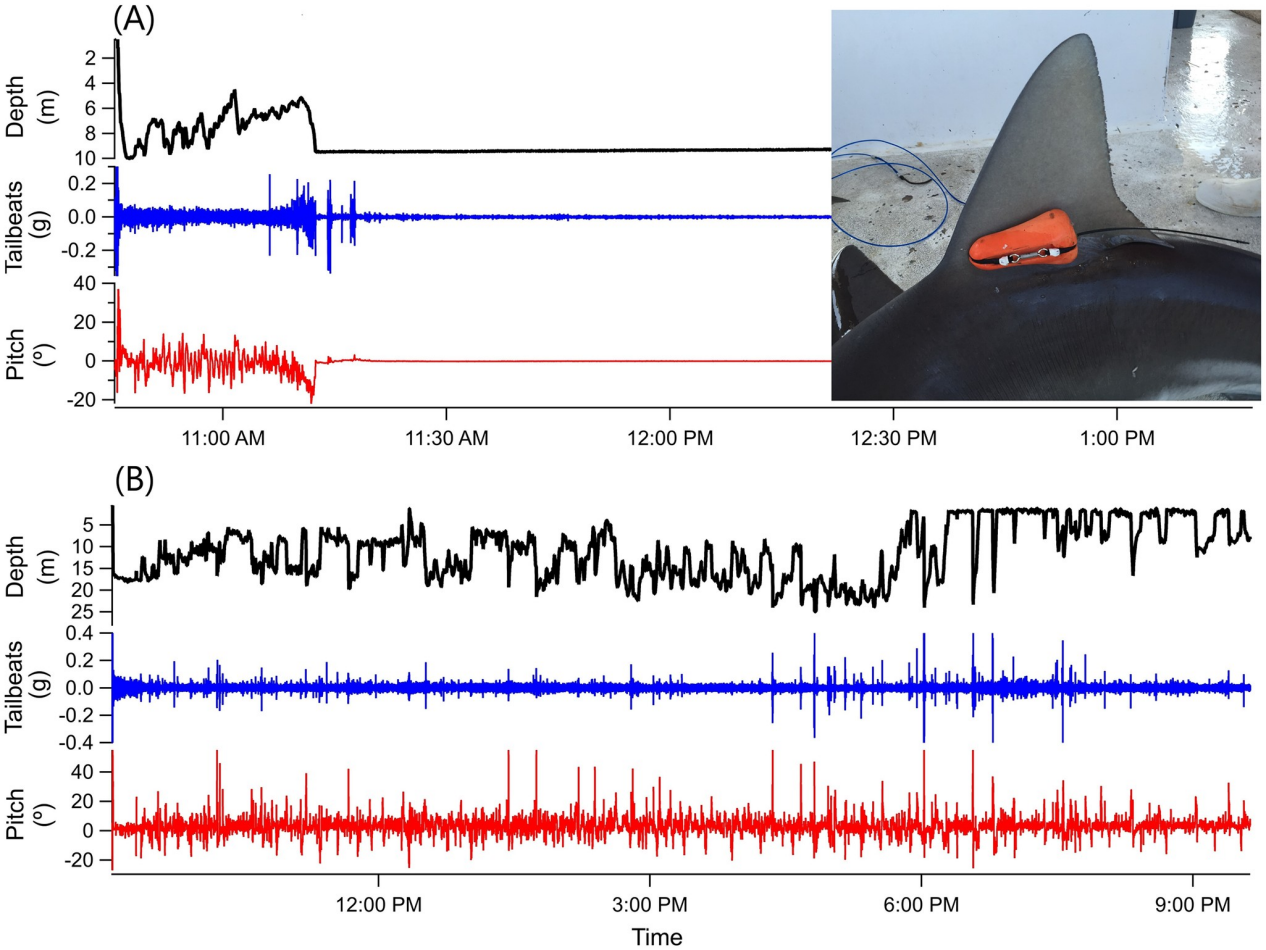
Acceleration traces depicting a post-release mortality (A) and post-release survival (B). Temperature, depth, pitch, and tailbeat movements for (A) a blacktip shark that died after release and (B) a sandbar shark that survived capture and release showing typical “yo-yo” diving behavior. The blacktip shark swam for 30 min before settling on the bottom in normal (dorsal side up) posture, and exhibiting erratic tailbeats for approximately five minutes until all movement ceased around 11:20 am. Inset shows a sandbar shark dorsal fin with ADL float package attached (Lear and Whitney 2016 [46]).

Cefas G6a+ ADLs (Cefas Technologies, Lowestoft, UK) were set to record triaxial acceleration at 25 Hz, depth at 1 Hz, and temperature at 0.03 Hz. ADLs were embedded in custom float packages alongside a VHF transmitter (Advanced Telemetry Systems, Isanti, MN, USA). These float packages were hydrodynamic, approximately 3 x 7 x 12 cm in size, and weighed 125 g in air (70 g positively buoyant in seawater, see [54]). This amount of positive buoyancy represents less than 0.5% of the body weight of tagged sharks, and is thus well below the 2% of animal body weight typically recommended for tagging studies [55,56]. Float packages were attached to the first dorsal fin of sharks at two points using a tether made from plastic cable ties or monofilament with a built-in galvanic timed release (International Fishing Devices Inc., Northland, New Zealand), which corrodes in seawater after a predetermined number of days (Fig 1), in this case ~1–5. Once the galvanic release dissolves, the tether releases, allowing the package to detach from the fin and float to the surface for recovery. Floating packages were detected using a hand-held, multi-channel VHF receiver (R45-20C, Advanced Telemetry Systems, USA), and physically recovered from a vessel following methods described by Lear and Whitney [46].

### Blood biochemistry analysis

Blood samples were assayed for pH onboard the fishing vessel within 10 min of the blood draw using an iSTAT-1 hand-held portable blood analyzer with CG4+ cartridges (Abaxis, Union City, CA, USA). Partial pressure of carbon dioxide and bicarbonate concentrations were also measured with the iSTAT, but are not reported here due to error associated with measuring these parameters at variable temperatures [57]. Values for pH were temperature-corrected to the mid-water temperature measured by the hand held meter using equation 1B from Mandelman and Skomal [37]. Hematocrit levels were also analyzed onboard following the blood draw using a hematocrit spinner (Zipocrit, LW Scientific, Lawrenceville, GA, USA). Additionally, 1–2 mL of the blood sample was spun down, separated into plasma and red blood cell layers, and immediately frozen using a liquid nitrogen dry shipper. Frozen plasma samples were later analyzed using bench-top Critical Care Xpress and pHOx blood gas analyzers (Nova Biomedical, Waltham, MA, USA) for glucose, lactate, and ion levels including potassium (K^+^), sodium (Na^+^), chloride (Cl^-^), magnesium (Mg^2+^), and calcium (Ca^2+^).

### Mortality rates and post-release recovery

At-vessel mortality rates were calculated by dividing the total number of individuals of each species landed dead by the total number of each species caught. In addition to the five species of large coastal sharks commonly caught in the Florida commercial shark longline fishery which were evaluated for PRM, AVM rates were also assessed for smaller species and other bycatch species including blacknose (*C*. *acronotus*), Atlantic sharpnose (*Rhizoprionodon terraenovae*), nurse (*Ginglymostoma cirratum*) and lemon sharks (*Negaprion brevirostris*).

Accelerometer data, analyzed using Igor Pro (v. 7.08; Wavemetrics, Inc. Lake Oswego, OR, USA), were used to determine mortality events. Mortality was easily distinguishable by a constant depth trace (on the bottom), and cessation of movement apparent in the acceleration traces (Fig 1). Time of death was determined as the point at which the shark settled on the sea floor. As monitoring durations and time to mortality were variable, we used the Kaplan-Meier method to estimate the survival function [58], with total PRM calculated as the proportion of mortality events identified across all individuals, and confidence intervals for PRM calculated using methods described by Goodyear [29]. Individuals that died after release were censored as a mortality at the time they settled on the seafloor, and individuals that survived until the ADL popped off were censored as a survivor at the time of pop-off. The probability of surviving was assessed for all species using this method. A two-way analysis of variance (ANOVA) was used to determine whether time to mortality varied between species. Additionally, for each species AVM and PRM were combined to provide an estimate of the total mortality rate for sharks that interacted with the gear, calculated as (1- *P*(surviving capture) *x P*(surviving post-release)).

Recovery periods were assessed for blacktip, sandbar, tiger, and bull sharks, for individuals that survived longline capture and had deployment periods greater than 12 h. Recovery periods were calculated using techniques described by Whitney et al. [45], who outlined several acceleration and depth-derived metrics for use in assessing recovery from capture. Here, tailbeat cycle (TBC) was chosen to determine recovery because it showed the most consistent, clearest relationship with time post-release across species. Tailbeat cycle was calculated using a wavelet transformation of the sway axis using the Ethographer extension [59] of Igor Pro. Calculated TBC was averaged into 10 min means for each shark, and these data were input into logistic models in R (v. 4.0.3; R Foundation for Statistical Computing, Vienna, Austria) to investigate relationships between time post-release and TBC, as described by Whitney et al. [45]. Time to recovery was calculated as the amount of time after release it took for TBC to gain 80 percent of the difference between the initial hour post-release and the fully recovered value, defined as the upper asymptote in the logistic equation. By definition, the recovered value can never fully reach the upper asymptote, and using an 80 percent threshold allowed for the calculation of a specific recovery time using consistent methodology.

### Predictors of at-vessel and post-release fate and recovery time

At-vessel metrics were examined for their potential to predict post-release fate of sharks for species with multiple mortalities. Logistic regressions built in R were used to determine relationships between either at-vessel fate (AVM or alive at vessel) or post-release fate (died or survived) and at-vessel metrics including blood biochemistry parameters, hook time, fish sex and size, reflex indices, and environmental conditions including water temperature and dissolved oxygen. All tested at-vessel metrics are listed in Supplementary Information, S2 Table. Since many at-vessel metrics were correlated with each other, logistic regressions with only one at-vessel predictor at a time were used rather than a larger model incorporating multiple at-vessel predictors to avoid problems associated with collinearity. Random effects of sampling site and sampling trip did not improve model fit, and therefore no random effects were included in the models. If the regression *p*-value for a metric was <0.01, the metric was determined to be a significant predictor of either AVM or PRM for that species. Following the determination of significant physiological predictors of mortality, relationships between the predictors that were typically significant across species were examined using linear regressions to further investigate the drivers of mortality in each species. Assumptions of all logistic and linear models were tested using diagnostic plots in R.

Additionally, decision trees were developed using the ‘rpart’ package in R [60] to determine threshold values of at-vessel metrics that could best predict post-release fate. Separate trees were developed for each species that showed multiple mortalities, and all at-vessel metrics (environmental conditions, morphological characteristics, reflex indices, and blood parameters) were provided as potential predictors. The prediction error of these trees was assessed using a jack-knife approach, where individuals from one sampling trip in turn were excluded from the dataset, a decision tree built from the remaining dataset, and the cut-off points indicated by the tree used to predict the fates of sharks caught in the excluded sampling trip.

Linear regressions were used to determine which at-vessel metrics were correlated with post-release recovery time. In this series of regressions, each at-vessel metric in turn was regressed against time to recovery in each species, again regressing each at-vessel metric individually against recovery time to avoid collinearity in regression predictors. Because random effects of sampling site and sampling trip did not improve model fit, they were not included in these regressions. If the regression *p*-value was <0.01, the metric was determined to significantly correlate with recovery period for that species. Assumptions of all models were tested using diagnostic plots in R.

## Results

Between December 2013 and November 2017, 70 longline sets were conducted, 54 near Madeira Beach, FL, 10 near Key West, FL and 6 off of Naples, FL. The gear was soaked between 2.2 and 17.5 h (mean ± S.D. 4.8 ± 2.9 h; Table 1), with sets ranging from 90 to 259 hooks (mean 198 ± 56). Sets were located between 1 and 38 km offshore (mean 15 ± 9 km), at depths ranging from 2 to 26 m (mean 12.1 ± 4.7 m) with water temperatures ranging between 15.0 and 31.9°C (mean 25.3 ± 4.3°C; Table 1).

**Table 1:** Sizes, water temperaures caught, hook times, and acceleration data-logger monitoring periods for all tagged animals.

Species	Total length (cm)	Water temp. (°C)	Hook time (min)	Soak time (h)	Monitoring period (h)
Sandbar shark *C*. *plumbeus*	200 ± 11 (162–227)	21.5 ± 2.6 (16.2–26.5)	208 ± 236 (3–891)	8.9 ± 4.2 (4.6–17.5)	17.3 ± 20.5 (1.3–216.5)
Blacktip shark *C*. *limbatus*	155 ± 15 (116–186)	28.0 ± 3.3 (18.8–31.3)	130 ± 161 (2–948)	4.9 ± 3.2 (2.2–17.5)	22.5 ± 14.3 (0.8–61.9)
Tiger shark *G*. *cuvier*	198 ± 35 (131–267)	22.4 ± 5.2 (16.2–31.3)	304 ± 273 (11–888)	10.0 ± 5.8 (3.4–17.5)	22.7 ± 11.1 (0.7–58.3)
Spinner shark *C*. *brevipinna*	190 ± 17 (143–211)	19.5 ± 3.5 (16.2–29.8)	397 ± 257 (44–785)	11.1 ± 5.3 (3.5–17.5)	19.1 ± 8.5 (12.6–31.9)
Bull shark *C*. *leucas*	222 ± 27 (181–269)	26.9 ± 3.9 (19.8–31.0)	245 ± 305 (34–956)	6.9 ± 5.3 (3.2–17.5)	49.2 ± 42.9 (3.7–137.5)
Blacknose shark *C*. *acronotus*	111 ± 4 (105–116)	24.4 ± 5.0 (18.8–31.0)	189 ± 207 (30–602)	7.7 ± 5.9 (3.1–16.3)	NA
All Species	189 ± 35 (105–352)	24.2 ± 4.5 (16.2–31.3)	221 ± 230 (2–956)	7.4 ± 4.7 (2.2–17.5)	21.7 ± 19.2 (0.7–216.5)

Monitoring periods are given only for animals which survived post-release. All factors are presented in mean ± SD, with the range given in parentheses.

A total of 928 sandbar, blacktip, tiger, spinner, bull, and blacknose sharks were captured. Of these, 488 had their blood sampled and analyzed, and 343 were tagged with ADLs (Table 2). Of the 343 ADLs deployed, we recovered and downloaded data from 316, a 92% data recovery rate. Tagged sharks ranged in size from 105 to 269 cm total length (Table 1), with girths ranging from 40 to 172 cm. Hook times ranged from 3 min to 15.9 h (mean 3.9 ± 4.0 h; Table 1), and handling time on deck ranged from 3 to 16 min (mean 5 ± 2 min).

**Table 2:** Species-specific catch numbers, at-vessel mortality (AVM), post-release mortality (PRM), and total mortality rates.

Species	Total caught	AVM	AVM rate (%)	*n* tagged	*n* used in PRM estimate	PRM	PRM rate (%)	Total mortality rate (%)
Sandbar shark	185	1	0.5	140	130	4	3.1 ± 2.5	3.6
Blacktip shark	292	102	35.0	110	105	44	41.9 ± 7.9	62.3
Tiger shark	126	0	0.0	55	52	1	1.9 ± 3.1	2.0
Spinner shark	55	34	61.8	17	14	10	71.4 ± 19.9	89.1
Bull shark	36	0	0.0	14	14	1	7.1 ± 11.3	7.1
Blacknose shark	234	50	34.9	7	1	1	(100.0)	(100)
All Species	928	207		343	316	61		

PRM rates are listed ± 95% confidence intervals, calculated using equations outlined by Goodyear (2002). Total mortality rates for each species were estimated using the probabilities of surviving at-vessel and surviving post-release using the equation specified in the text. Mortality rates in parentheses are based on a single shark. Unrecovered ADL floats were not included in post-release mortality rate calculations.

### At-vessel mortality

At-vessel mortality rates varied substantially by species. Tiger and bull sharks showed 0% AVM and sandbar sharks showed 0.7% AVM. The single AVM for sandbar sharks had a hook time over 12 h, although several other sandbar sharks with similar hook times were landed alive. Blacktip, blacknose, and spinner sharks had higher levels of AVM (47–67%; see Table 2). For these species, AVM was composed of two parts, death from capture and death from depredation while on the line. The portion of AVM due to depredation was 12%, 26%, and 3%, for blacktip, blacknose, and spinner sharks, respectively (n = 12, n = 12, and n = 1 depredations, respectively). In addition to the main species investigated in this study, nurse (n = 213), lemon (n = 6), and Atlantic sharpnose sharks (n = 46) were also caught, and showed AVM rates of 0% for nurse and lemon sharks, and 67.4% for Atlantic sharpnose sharks.

Several factors were found to correlate with AVM rates. Different metrics were significant predictors of AVM for different species (Table 3), but there were some similarities across species. For example, time on the line and blood pH, lactate, K^+^, and Cl^-^ were significant predictors of AVM for all species with observed AVMs in which blood chemistries were measured (blacktip, blacknose, spinner, and sandbar sharks). Sex and size did not significantly affect AVM rates in any species. Several hook timers also showed times indicating that they were triggered during deployment of the longline, potentially by catching on benthic structure during deployment. If these hook timers were excluded from analyses, logistic models indicated that spinner sharks had a 50% percent chance of being an AVM after 5.1 h on the line, blacktip sharks at 4.9 hours, and blacknose sharks at 2.8 h. No other species showed multiple AVMs. Mean values for all at-vessel metrics for AVMs and individuals alive at vessel for each species are provided in S3 and S4 Tables.

**Table 3:** Comparisons of at-vessel metrics between individuals alive and dead at capture, and individuals that survived or died post-release.

Species	AVM vs. Alive at vessel		PRM vs. Survived	
Sandbar shark *C*. *plumbeus*	K^+^	*p*<10^−11^	K^+^	*p*<10^−6^
	Lactate	*p*<0.001	Lactate	*p*<10^−5^
	Mg^2+^	*p*<0.001	pH	*p*<0.0001
	Cl^-^	*p*<0.01	Mg^2+^	*p*<0.001
	(pH)	*p*<0.05	Cl^-^	*p*<0.01
	(Time on line)	*p*<0.05	Release condition	*p*<0.01
	(Glucose)	*p*<0.05	NM reflex	*p*<0.01
			(Time on line)	*p*<0.05
Blacktip shark *C*. *limbatus*	pH	*p*<10^−15^	pH	*p*<10^−7^
	K	*p*<10^−15^	K^+^	*p*<10^−5^
	Lactate	*p*<10^−8^	Lactate	*p*<10^−5^
	Time on line	*p*<10^−6^	Release condition	*p*<0.0001
	Ca^2+^	*p*<10^−5^	Equilibrium reflex	*p*<0.01
	Mg^2+^	*p*<0.0001	Mg^2+^	*p*<0.01
	Cl^-^	*p*<0.001	(Cl^-^)	*p*<0.05
	(Hematocrit)	*p*<0.05	(Ca^2+^)	*p*<0.05
	(Total length)	*p*<0.05	(DO)	*p*<0.05
	(Na^+^)	*p*<0.05	(Water temperature)	*p*<0.05
Tiger shark *G*. *cuvier*	NA		(Cl^-^)	*p*<0.05
Spinner shark *C*. *brevipinna*	pH	*p*<10^−11^	Release condition	*p*<0.01
	K^+^	*p*<10^−6^	(Lactate)	*p*<0.05
	Time on line	*p*<10^−5^		
	Cl^-^	*p*<10^−5^		
	Na^+^	*p*<0.0001		
	Lactate	*p*<0.0001		
	Mg^2+^	*p*<0.001		
Bull shark *C*. *leucas*	NA		(K^+^)	*p*<0.05
			(pH)	*p*<0.05
Blacknose shark *C*. *acronotus*	pH	*p*<10^−10^	NA	
	K^+^	*p*<10^−8^		
	Time on line	*p*<0.0001		
	Cl^-^	*p*<0.001		
	Lactate	*p*<0.001		
	Ca^2+^	*p*<0.01		

The at-vessel metrics, including morphological and behavioral measurements, physiological blood parameters, environmental conditions, and time on the line, which showed significant differences (logistic regression *p*<0.01) between individuals alive at vessel versus at-vessel mortalities (AVM), and individuals that survived after release versus post-release mortalities (PRM). All metrics with a significant *p*-value are listed, in order of decreasing significance, as well as those with p<0.05 in parentheses which show non-significant trends. Significance was assessed using logistic regressions for each parameter per species individually, as at-vessel metrics showed high collinearity.

### Post-release behavior and survival

**Fig 2:**
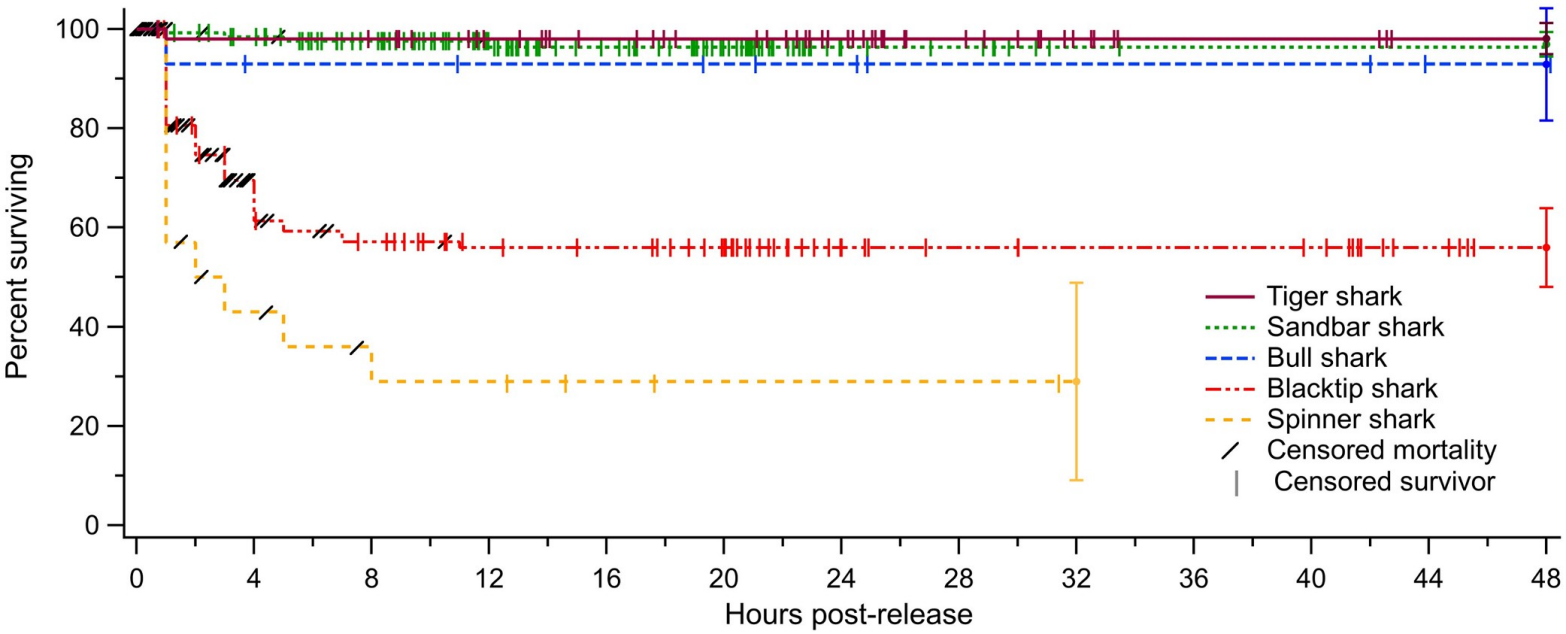
Kaplan-Meier survival curves for the five species of large coastal sharks tagged in the current study. Traces show the probability of mortality for the first 48 hours post-release for each species (spinner sharks were only monitored up to 31 hours post-release). The majority of mortalities occurred within the first hour post-release, and all mortalities occurred within 12 hours post-release. The error bars at the end of each curve show the 95% confidence intervals of the final estimated mortality rate for each species, calculated using equations set by Goodyear (2002).

Live sharks were released and monitored with animal-borne ADLs for periods ranging from 0.7 to 205 h (mean 20.9 ± 19.6 h), collecting more than 6,400 h of fine-scale acceleration data in total. Out of the 316 recovered accelerometers, we observed 61 PRM events (Table 2). Sharks that died typically showed irregular diving patterns before settling on the bottom, at which time they typically maintained normal (dorsal side up) body orientation and showed erratic tailbeats for a short time before they ceased all tailbeat movement (Fig 1A). Sharks that survived post-release typically showed repetitive oscillations, or “yo-yo” diving behavior, between the surface and sea floor for the majority of the deployment, with corresponding changes in body pitch during the dive cycle and consistent tailbeat patterns (Fig 1B). All observed mortalities occurred within 12 hours of release, with the median time to mortality being 1.23 h. The majority of mortalities (59%) occurred within 2 h of release, and over 90% occurred within 5 hours (Fig 2). There was no difference between species in time to mortality post-release (ANOVA F = 2.36, p = 0.11).

Several (n = 12; 20%) of the PRMs were scavenged after death and their tags ingested, as evidenced by acceleration data from a stationary tag on a dead animal suddenly showing erratic movements, followed by a consistent, unnatural tag orientation and a slower tailbeat cycle than that of the originally tagged shark (indicating that the tag was in the stomach of a larger fish; see [46]). Many other mortalities also appear to be scavenged before the tag released from the animal (but without the tag being ingested), evident by large erratic acceleration movements observed on dead animals. Ingested tags were regurgitated between 0.2 and 30 days later (mean 6.0 ± 7.7 days). Additionally, one blacktip shark appears to have been directly predated (and its tag ingested) while the animal was alive (3.4 hours after release), as identified by the same changes in acceleration data described for scavenging, except with the changes initiated while the tagged animal was still swimming instead of after it had come to rest on the bottom.

**Fig 3:**
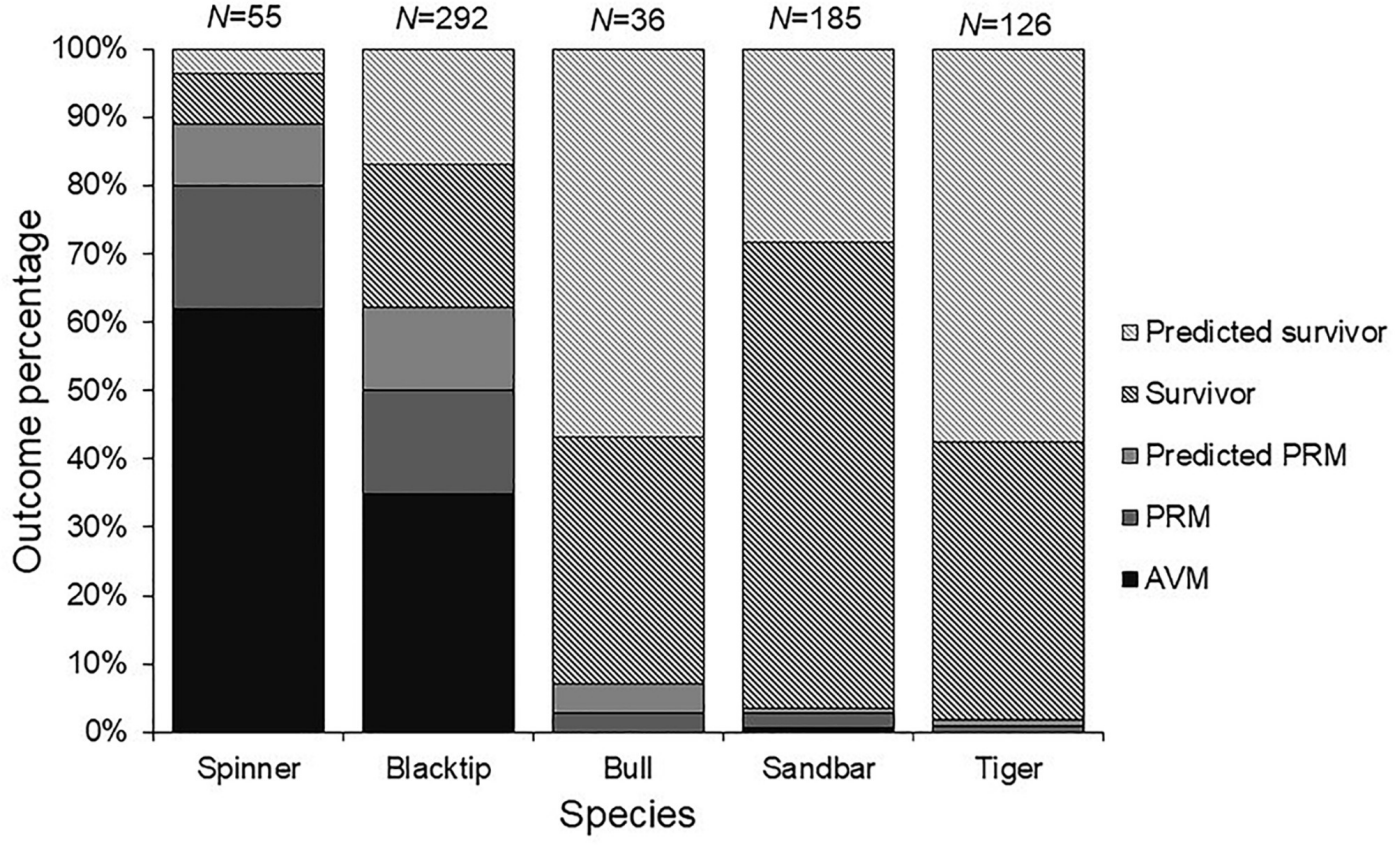
At-vessel and post-release fate percentages of the total catch by species. Individuals with the fate unknown (released alive but not tagged) were separated into either a predicted post-release mortality or predicted survivor by mirroring mortality rates for individuals with known outcomes. AVM = At-vessel mortality, PRM = post-release mortality.

Overall PRM rates varied substantially by species, but were separated into two groups. Sandbar, tiger, and bull sharks had low PRM rates of 3.1%, 1.9%, and 7.1%, respectively, whereas blacktip and spinner sharks had significantly higher PRM rates of 41.9% and 71.4%, respectively (Table 2). All species had higher PRM rates compared to AVM rates (PRM rates ranged from 1.8–9.6% higher than species-specific AVM rates). Total fishing mortality was high for blacktip and spinner sharks (62.3% and 89.1% respectively), but sandbar, tiger, and bull sharks all experienced total fishing mortality rates under 10% (Table 2; Fig 3).

### Correlating at-vessel metrics with post-release fate and recovery period

Several at-vessel metrics showed significant relationships with post-release fate, but most metrics also showed a large amount of overlap between sharks that survived and sharks that succumbed to mortality. Similar to AVM, different metrics were significantly correlated with PRM for different species (Table 3), although there were a few similarities. For example, as with AVM, blood pH, lactate, K^+^, and Cl^-^ showed a significant correlation with PRM for blacktip and sandbar sharks (logistic regression p<0.01). It is also notable that while water temperature showed only a non-significant trend with PRM in blacktip sharks (the only species with high mortality rates caught at a wide range of water temperatures) as a continuous predictor, rates of PRM varied substantially when considered in high temperatures (e.g. at >27°C PRM rate = 49 ± 0.5%) compared to lower temperatures (at <27°C PRM rate = 29 ± 0.5%). The only metric significantly correlated with PRM for spinner sharks was release condition, with lactate showing a non-significant trend (p<0.05). In bull and tiger sharks only one PRM was identified for each species; in tiger sharks the one PRM was foul-hooked in the pectoral fin. No at-vessel metrics were significantly correlated with PRM in either species, although K^+^ and pH showed nonsignificant trends in bull sharks and Cl^-^ a non-significant trend in tiger sharks (logistic regression p<0.05). Reflex indices overall were not well correlated with PRM, with only nictitating membrane reflex in blacktip sharks and equilibrium reflex in sandbar sharks emerging as low-level significant predictors of mortality (Table 3). However, release condition was a significant predictor of PRM for blacktip, sandbar, and spinner sharks, with all blacktip and spinner sharks with a release condition of 4 (‘poor’ swimming ability, including weak, erratic, or absent tailbeats and/or an inability to uphold equilibrium, see S1 Table) dying post-release, although some individual sandbar sharks with this release condition survived post-release. Mean values for all at-vessel metrics for PRMs and surviving individuals for each species are provided in Supplementary Information, S3 and S4 Tables.

**Fig 4:**
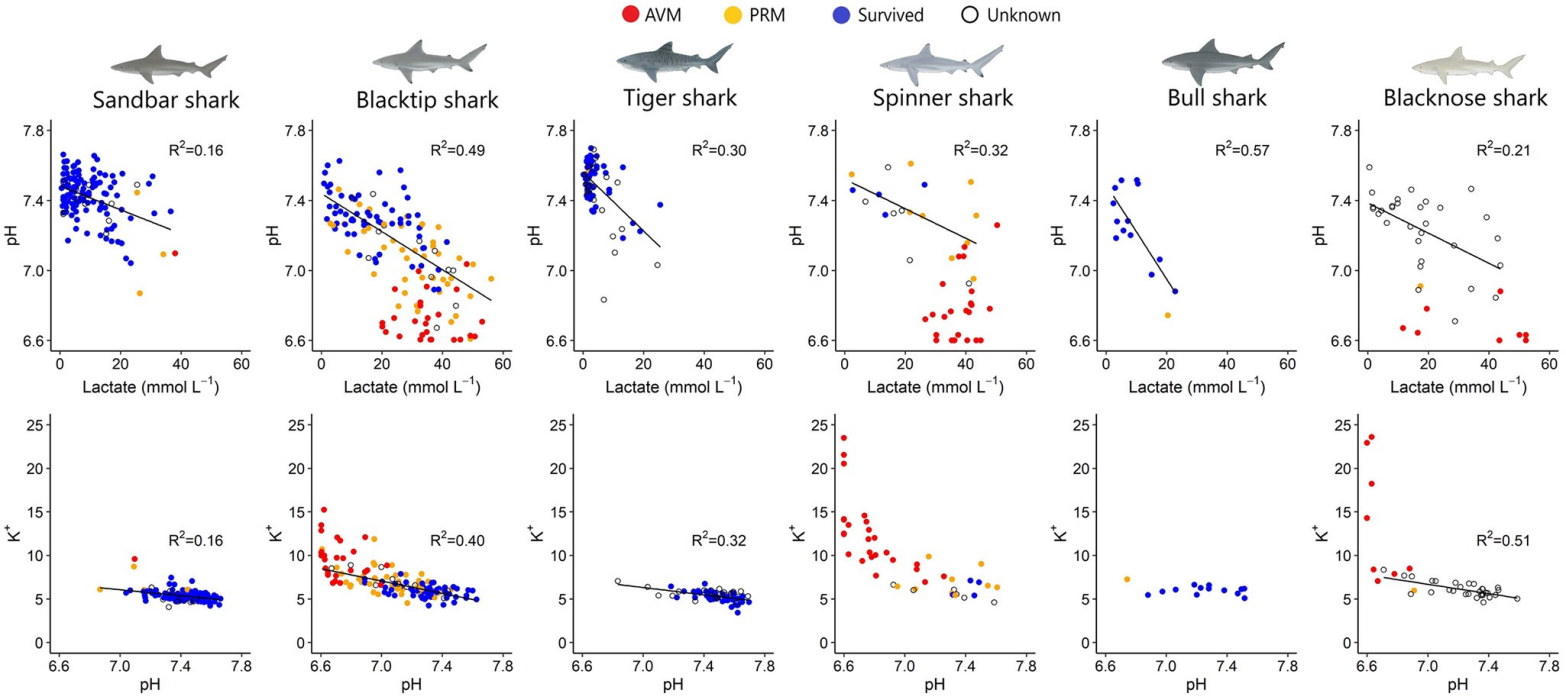
Relationships between blood parameters describing the reaction to capture stress experienced by each species of shark. Linear trendlines are drawn where significant relationships between parameters were determined. Data from all sharks where blood was taken were included in the plots, including from sharks that were at-vessel mortalities (AVM), post-release mortalities (PRM), sharks that survived the capture process (Survived) and sharks which were alive at vessel but with the post-release fate unknown (Unknown). However, only sharks that were alive at the time of capture were used to test significance of relationships between parameters and form trendlines as it was unknown how long AVM sharks had been dead at the time of sampling.

As pH, lactate, and potassium were typically among the physiological factors most predictive of mortality and are also important indicators of physiological processes such as acidosis and homeostasis responses, linear regressions were run between these parameters in each species to further investigate the drivers of observed mortality or physiological disruption. These regressions showed significant negative linear relationships between pH and lactate in all species (Fig 4). Additionally, pH and K^+^ were significantly negatively correlated in all species except for spinner sharks (Fig 4).

Behavioral recovery from capture based on fine-scale swimming patterns was assessed for species with more than five tagged individuals surviving release. Individual recovery periods for sharks ranged from 3.4 to 25.9 h. Average species-specific recovery periods were 11.7 ± 4.6 h for sandbar sharks, 11.5 ± 5.1 h for blacktip sharks, 10.6 ± 4.7 h for tiger sharks, and 12.9 ± 7.5 for bull sharks. No at-vessel metrics were significantly correlated with time to recovery in any species, but non significant positive trends with time to recovery (linear regression p<0.05) were identified for lactate in sandbar sharks, water temperature in blacktip sharks, and time on line and release condition in tiger sharks.

### Predicting post-release fate from blood stress physiology

**Fig 5:**
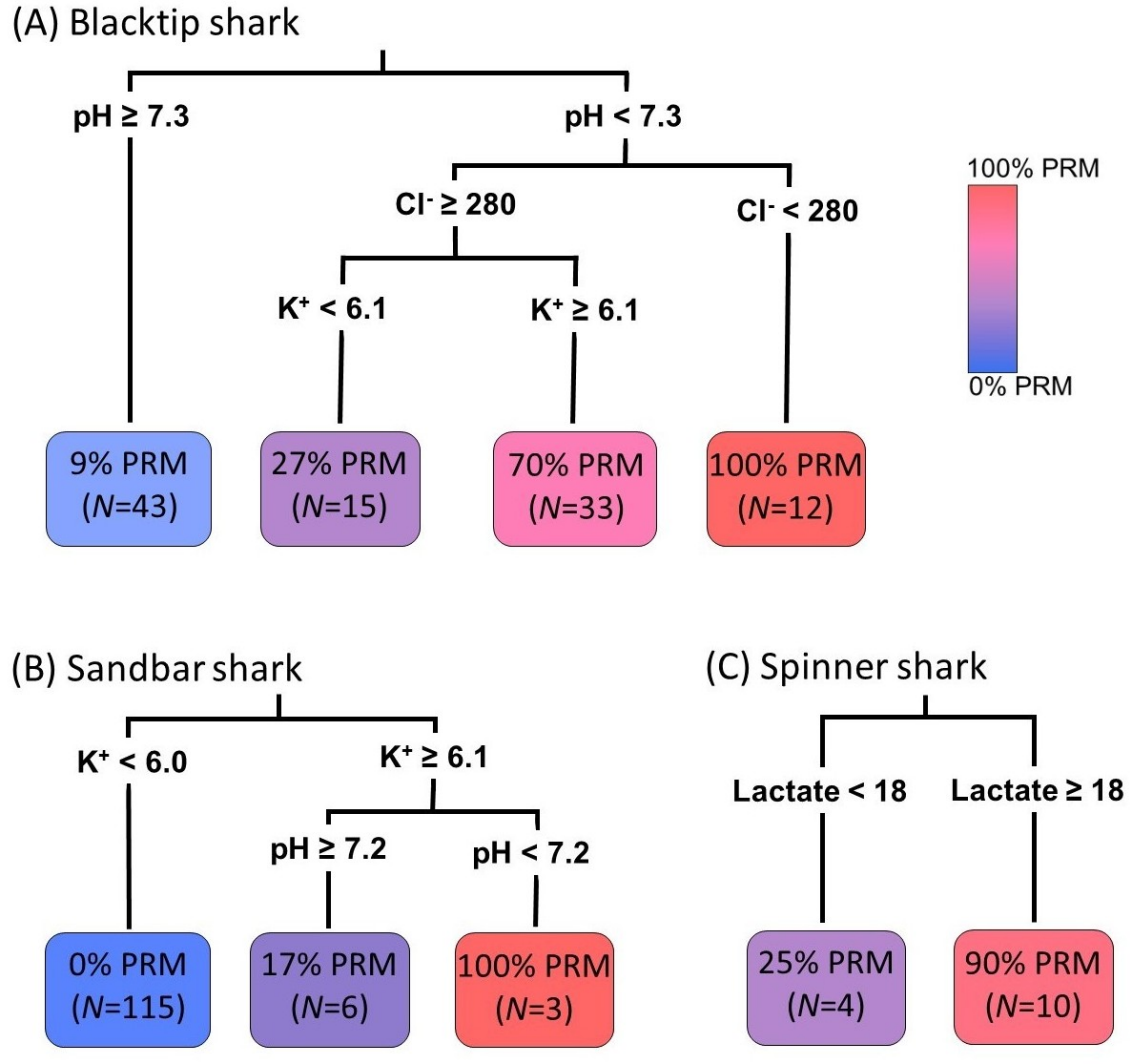
Decision trees using at-vessel metrics to predict post-release outcomes for (A) blacktip, (B) sandbar, and (C) spinner sharks.

When at-vessel metrics were input into the formation of decision trees to determine threshold values that predict PRM for each species, only pH, K^+^, and Cl^-^ were maintained as informative predictors for mortality in blacktip sharks, pH and K^+^ for sandbar sharks, and lactate for spinner sharks (Fig 5). However, because of the large overlap in measured at-vessel metrics between PRMs and sharks that survived, there were not always distinct breakpoints in these parameters that indicated mortality. As a result, the regression trees showed only moderate accuracy in some species. Jack-knife error testing of trees for sandbar sharks resulted in 99% prediction accuracy (128 of 129 fates predicted correctly), but only 68% prediction accuracy in blacktip sharks and 69% prediction accuracy in spinner sharks. However, for blacktip and spinner sharks the rate of false positive and false negative predictions was approximately equal and, as a result, the total predicted mortality rate for blacktip sharks had only 2% error compared to the measured mortality rate, and the total predicted mortality rate for spinner sharks had 8% error from the measured mortality rate.

## Discussion

Studies of PRM in sharks are imperative for accurate stock assessment and management, but are logistically difficult and often limited in sample size. Our application of re-usable ADL tags allowed us to monitor over 300 sharks from five species, providing reliable estimates of PRM in a commercial longline fishery. The collection of blood from each of these tagged sharks, as well as from over 600 untagged individuals, allowed us to evaluate the relationship between hematological blood-stress indicators and post-release fates. Directly pairing blood-stress indicators with animal fates for a large sample size provides the most robust analysis to date of shark physiological metrics on PRM.

### At-vessel mortality

The AVM rates measured in this study should be considered conservative estimates, as some soak times were intentionally shortened in order to target live animals for our main purpose: the study of PRM. However, the relative species-specific AVM rates were similar to those reported in past studies, with the order of vulnerability to AVM increasing from nurse, lemon, tiger, and bull sharks, with low mortality rates (0–15% AVM reported previously [61–64]), to sandbar sharks which typically show intermediate mortality rates (17–36% AVm reported previously [61–63]), to blacknose, blacktip, spinner, and Atlantic sharpnose sharks, which have demonstrated AVM rates between 80 and 100% in previous studies [61–64].

### Post-release mortality rates

Although few previous studies have directly assessed shark PRM in a BLL fishery, the high variability of PRM rates determined in this study emphasizes the importance of assessing PRM on a species-specific basis. In sandbar sharks, our measured PRM rate of 3.3% (N = 130) is lower than that estimated by both Marshall et al. [25] of 20% (*N =* 10) in the NW Atlantic, and Barnes et al. [65] of 25% (*N* = 8) off the southeast coast of Australia. The average hook times of sandbar sharks tagged by these previous studies were slightly longer (224 min and 419 min by Marshall et al. [25] and Barnes et al. [65], respectively, compared to 208 min in the present study), with few PRM events observed in sharks on the line for <3 h [25], and most mortalities observed in sharks hooked for >7 h. However, differences in hook times cannot fully account for the discrepancy as, in the present study, the PRM rate for sandbar sharks hooked for >7 (n = 29) was only 6%, compared to 33% (1 of 3 individuals hooked for >7 h) found by both Marshall et al. [25] and Barnes et al. [65]. Other factors related to differences in handling of sharks (e.g. Barnes et al. [65] also surgically implanted acoustic transmitters and Marshall et al. [25] did not irrigate sharks while onboard) or fishing conditions or methods (e.g. water temperature, gangion length, etc.), as well as the relatively small sample sizes of the previous two studies may contribute to the discrepancy.

Few published studies have assessed PRM rates in tiger, bull, blacktip, or spinner sharks following fisheries interactions. However, our finding of a 2.0% PRM rate in tiger sharks is similar to estimates from drumline fishing methods in the NW Atlantic (n = 28 [39]) and bottom longline gear in the SW Atlantic (n = 21 [15]), where no capture-related mortality was reported in tiger sharks. The estimate of 26% PRM reported for bull sharks on drumline gear in the NW Atlantic (n = 27 [39]) is over three times as high as our study, although this previous estimate was based on reporting rates of fin-mounted satellite tags and may overestimate mortality if tag failure or shark behavior prevent satellite transmissions. In blacktip sharks, PRM rates have previously been estimated at 9–23% in recreational fisheries [17,27,44], and we found substantially higher PRM for this species in the BLL fishery.

As an alternative to empirically derived animal fates, several past studies have investigated the theoretical susceptibility to PRM for shark species based on their AVM rates and relative levels of physiological disturbance analyzed through blood samples. Although such studies cannot directly assess mortality rates, they have proposed a relative susceptibility to mortality where tiger and bull sharks are the least vulnerable, sandbar sharks show intermediate physiological disturbance, and blacktip sharks show high levels of disturbance [37,39,40]. This relative order of vulnerability to capture stress is supported by the PRM rates in the present study, with the exception of bull sharks having a higher PRM rate (7%) than sandbars (3%), although the small sample size of bull sharks in this study (*N* = 14) introduces wider margins of error (±11%) for the PRM rate of this species.

While PRM rates varied substantially between species, they were consistently higher than AVM rates (by approximately 2–10% depending on the species). Previous studies have found similar results, with PRM rates 6% higher than AVM rates in blue sharks [20], 7% higher in dusky sharks [25], and 15% higher in sandbar sharks [25]. Previously, this difference in PRM and AVM rates has been used by fisheries managers to estimate PRM rates in the absence of empirical PRM data [e.g. 66] by assuming that PRM rates are 6% higher than observed AVM rates based on work by Campana et al. [20] in blue sharks. Our findings support this as a viable way to approximate PRM when empirical measurement of post-release fate is not possible. This may also be a valuable way to assess whether a PRM rate determined for a species in one population or under one set of fishing conditions can be applied to different regions or fisheries. If the AVM rates recorded between two studies are approximately equal, applying the PRM rate estimated under one set of circumstances to the other may be a viable option. Regardless, given the interspecific variation in the magnitude of the difference between AVM and PRM in this and other studies and the variation within a species between studies (e.g. Marshall et al. [25] found PRM 15% higher than AVM in sandbar sharks, but the present study found PRM only 2.5% higher than AVM for the same species), empirically deriving estimates of PRM is always preferable.

Although our monitoring period of tagged animals (overall mean 20.9 h) was shorter in duration than what is typically sought in PRM studies using satellite tags (weeks) or passive acoustic telemetry (months to years), the majority of PRMs of longline-caught sharks occur immediately after release [14], and many previous studies documented all mortalities within a few days of release, even with monitoring periods of up to >200 d [e.g. 18,25,28,65,67,68]. Additionally, recovery period analysis shows behavioral recovery from capture stress in an average of 10–13 h, depending on the species, recovery times that are mirrored by studies in the laboratory demonstrating blood-based physiological recovery from capture in 6–24 h [16,69] and metabolic recovery from longline capture within 12 h [70], although capture methods and durations are highly varied in these previous studies. However, delayed mortality of a few days up to 135 days post-release has been reported for some pelagic [14,19,20,23,24,26,71] and deep water [72] shark species, but in most cases it is unclear whether long-delayed mortalities can be attributed to the capture event or to natural factors [11,26]. Given our sample size and the large number of surviving sharks monitored for at least twice as long as the time of the latest PRM, rare delayed mortalities would have a negligible effect on our calculated rates for all species except spinner sharks. Low sample size and high mortality rate could mean that our results underestimate PRM rates in spinner sharks.

### Physiological correlates of mortality

The magnitude of the physiological response to capture in elasmobranchs is mediated by several factors which can vary substantially between species and individuals based on life history, ontogenetic stage, environmental conditions, and health status [73]. These factors include (1) the magnitude of the endocrine stress response, (2) aerobic capacity, which governs the degree of respiratory acidosis, (3) anaerobic capacity, which governs the degree of metabolic acidosis, (4) the behavioral response to capture, i.e. the degree of struggle or ‘fight’ when hooked, which affects aerobic and anaerobic costs, and (5) the ability of a species or individual to recover following disruption of physiological homeostasis, which is likely linked to aerobic scope and ventilation strategy [8,9,37,39,40,43,73–75]. While the initial stress response is meant to trigger and support a fight-or-flight response, prolonged acute stress events may result in a physiological state that is quite harmful to normal cellular functions. These physiological repercussions include the release of high levels of catecholamines or corticosteroids from the primary stress response, respiratory acidosis caused by high levels of carbon dioxide built up in the blood due to inadequate ventilation on the line, and/or metabolic acidosis caused by high levels of lactic acid built up in the blood due to anaerobic muscle work [9,73,74].

In the present study, several physiological indicators can provide insight into the interspecific stress response, with potential clues about how the pathways described above may be driving PRM. For sandbar and blacktip sharks, pH was the most influential factor for predicting PRM, and across blacktip, sandbar, spinner, and blacknose sharks, there were significantly decreased pH levels in sharks dead upon capture compared to those alive at the boat. Additionally, pH levels of most captured animals were substantially lower than what is thought to be the typical baseline pH of elasmobranchs (7.7–8.0 [37]). The one PRM observed in bull sharks also recorded the lowest pH value (6.7) of all tagged bull sharks. Together, these factors show that there is substantial acidosis occurring in all species in response to the capture process, which is likely a main cause of mortality in blacktip, sandbar, spinner, blacknose, and possibly bull sharks. This has also been suggested by previous studies showing significant declines in pH in longline-caught blacktip, sandbar, tiger, Atlantic sharpnose, dusky, and Caribbean reef sharks [e.g. 9,37,39,42,73,74]. For all species studied here, pH showed a significant negative relationship with lactate, indicating metabolic acidosis, but it is also likely that a degree of respiratory acidosis is occurring, although this cannot be confirmed from our methods. A combination of metabolic and respiratory origins has been previously suggested in tiger and sandbar sharks [37], although acidemia observed in blacktip sharks has been attributed to mainly respiratory causes [37], while acidemia in Atlantic sharpnose, dusky, and blue sharks has been proposed to be mainly metabolic [37,74].

In addition to pH, our results indicate that potassium concentrations were also an important predictor of mortality in blacktip and sandbar sharks. Potassium levels were also higher in spinner and blacknose sharks dead at vessel than those alive at vessel (S4 Table), and the one bull shark PRM observed, in addition to having the lowest measured pH, had the highest measured potassium level of all tagged bull sharks (7.2 mM). Previous studies have documented elevated potassium levels in sharks in response to gillnet or longline capture [e.g. 37,40,42,43,68,69,76], but were unable to draw a direct relationship to post-release fates. Dapp et al. [77] also observed a significant increase in potassium in bronze whaler sharks (*C*. *brachyurus*) that were either moribund or dead at the time of capture (i.e., at-vessel conditions). Elevated potassium levels in the blood can be the result of cell damage or a response to acidosis in which cells transfer cations (including K^+^) out of muscle cells in an effort to maintain blood pH and electroneutrality [73]. The association between potassium and pH observed in blacktip, sandbar, tiger, and blacknose sharks in this study (Fig 4) suggests that this latter pathway may be occurring in these species. Once in the blood, elevated potassium alters electrochemical gradients and can impact function of the heart and skeletal muscle, causing bradycardia, myocardial infarction, and neuromuscular interference [73], but more work is needed to fully understand the impact of elevated plasma potassium on these fishes.

Interestingly, sodium (Na^+^), the only monovalent electrolyte other than potassium, was not correlated with mortality in any species. Additionally, sodium did not significantly correlate with pH in any species except for tiger sharks (*p*<0.001, R^2^ = 0.14). As a monovalent ion and the most prevalent positively charged inorganic salt in fishes [78], sodium would theoretically also be transferred into the blood in high levels to maintain electroneutrality following intracellular acid-base imbalance, but it appears that this is not the case in most species studied here. However, for several of the species studied here, we see mortality associated with declines in blood Cl^-^ (S4 Table). In many taxa including fish, Cl^-^ is coupled to ${\text{HCO}}_{3^{-}}$ exchangers that can be affected by acidotic events [79,80]. The relationship between pH, K^+^, Cl^-^, and ${\text{HCO}}_{3^{-}}$ transporter response to acidosis has been well studied in mammals, but many of these isoforms have been identified in fishes as well [80,81]. Most research on acid-base balance in fishes has focused on expression of ion transporters in gill cells of fishes [reviewed by 81], but more work is needed to untangle associations between Na^+^, K^+^, Cl^-^, pH, and ${\text{HCO}}_{3^{-}}$ transport in other tissues, and how these connect to stress-induced mortality.

### Interspecific differences and predicting mortality

The similar relationships between physiological parameters and the fact that similar parameters were associated with PRM across species here suggest that physiological drivers of mortality may be shared across the species assessed in this study. Additionally, our results suggest that different mortality rates between species in our study may not be due to differential physiological stress responses between species or a differential ability to return to homeostasis following a given level of physiological disruption, but may instead be due to interspecific differences in physiology and behavior that regulate the magnitude of physiological disruption following capture. This hypothesis is supported by the similar cut off points between blacktip and sandbar sharks for the levels of pH and potassium that indicate mortality (pH = 7.2/7.3 and K^+^ = 6.0/6.1 for sandbar/blacktip sharks, respectively). This suggests that the substantially higher mortality rate observed in blacktip sharks compared to sandbar sharks may not be due to sandbar sharks having the ability to recover from greater physiological disruption, but to blacktip sharks reaching much higher levels of disruption during the capture process (see Supplemental S3 and S4 Tables). However, this was not the case in all species. For example, several bull and tiger sharks showed pH values lower than 7.2 and potassium concentrations higher than 6.1, but survived after release, as did some individual sandbar and blacktip sharks.

As the level of acidosis attained during capture (i.e. degree of pH and potassium imbalance) appears to be a main driver of PRM in most species in this study, factors that determine the level of acidosis experienced for species or individuals are likely to play a large part in determining mortality. For example, sharks that struggle or fight more on the line will build up greater levels of both CO_2_ and lactate in the blood through higher aerobic and anaerobic work, causing greater acidosis. Several previous studies have also hypothesized that species which struggle more intensely on lines create greater physiological disruption and are more susceptible to mortality [e.g. 82–85]. Previous research has shown that blacktip sharks fight much more strongly and for a greater duration on longlines compared to tiger sharks and nurse sharks [83]. Comparatively, sandbar sharks have been observed to cease fighting and lie on the bottom when captured on drumlines (Authors, unpublished data), suggesting that this species may have a more subdued response to capture or is capable of buccal pumping for short periods to recover from exhaustion. This high fight response for blacktip sharks and relatively calmer response of sandbar and tiger sharks may be a substantial driver of interspecific differences in mortality rates observed in this and previous studies. Similarly, water temperature is likely to influence the degree of acidosis experienced, as higher metabolic rates [86] and activity levels [87] of ectothermic sharks at warmer water temperatures would lead to higher levels of both respiratory and metabolic acidosis. This could account for the substantially higher PRM rates in blacktip sharks at warmer water temperatures observed here. This finding has been mirrored in studies in teleosts [88], for example, several studies have determined that rates of post-release mortality in Atlantic salmon (*Salmo salar*) increase significantly at high water temperatures [89–91].

In addition to differences in the level of physiological disruption observed between species, there was also a high amount of intraspecific variation in the relationships between blood stress indicators and mortality, making it difficult to provide a set of clear at-vessel predictors of mortality for some species. This was apparent through the relatively high rate of incorrect individual fates predicted for blacktip and spinner sharks using decision trees made with at-vessel measurements (67–69% of fates correctly predicted). However, at-vessel decision trees proved highly effective at predicting sandbar shark fates (>99% correctly predicted), and for blacktip and spinner sharks the predicted overall mortality rate of the sample was still relatively accurate, indicating that these types of tools could be a sufficient method of predicting overall mortality of sharks once blood data are calibrated to animal fates. Although the trees for sandbar and blacktip sharks identified fairly similar mortality thresholds, wholly different parameters were identified for spinner sharks, and it is unclear whether any tree built for a single species could be accurately applied to another species. Therefore, the mortality threshold metrics and values would have to be investigated for individual species to use such predictive tools with any confidence.

### Implications and recommendations for fisheries management

The results of this study highlight several factors relevant to common management measures in commercial fisheries. For example, our findings suggest that no-take regulations, if implemented, would likely be beneficial for robust species including sandbar, tiger, and bull sharks which suffered limited PRM, but would be less effective for blacktip and spinner sharks, which are much more susceptible to both AVM and PRM. This is particularly apparent for spinner sharks, of which nearly 90% would still have died in this study if all were released. For no-take regulations to be effective for these two species, soak times would have to be limited to approximately 5 h to reduce PRM by 50%. Although longlines are often soaked for >12 h during commercial fishing operations [92], shorter soak times may not substantially reduce catch rates, as comparisons of hook time and soak time of sets deployed for >6 h in this study show that the majority (>50%) of sharks were hooked within an hour of setting, with >70% caught within 3 h of setting, similar to the results of Marshall et al. [25]. Findings that blacktip shark mortality rates were substantially higher at warmer water temperatures suggest that seasonal restrictions or soak time limitations could also alleviate mortality in this species. Overall, the high PRM rates of some species make it essential that these data are incorporated into stock assessments and harvest quotas for sustainable shark fisheries. Additionally, the high variation in mortality rates between species and studies emphasizes the importance of quantifying PRM rates for the specific species and fishery in question.

## Supporting information

S1 Table Description of how condition index scores were assigned for released sharks. Guidelines were first set by Hueter et al., (2006) [53].
(PDF)

S2 Table All at-vessel metrics used in analyses. Metrics are divided by categories, which are also referred to in the text.
(PDF)

S3 Table At-vessel measurement results from each species of shark sampled depending on at vessel condition or post-release fate. PRM = Post-release mortality; AVM = at-vessel mortality. Values are presented as mean ± SD. Reflexes (nictitating membrane (NM), flex, bite, and equilibrium) were scored as a ‘1’ if unimpaired and a ‘0’ if impaired or absent, and release condition was scored as 1 to 4 based on swimming ability, with 1 indicating strong ability and 4 poor ability. The numbers of animals in each category (*N*) are included, but note that this sample size is not necessarily the same for all parameters reported, as certain parameters were not necessarily available for all individuals, particularly for AVM and Alive at vessel individuals that were not tracked post-release. An ‘*’ after the AVM value and blue shading indicates a significant difference (logistic regression *p*<0.01) of metric values between AVM and Alive at vessel individuals, and a ‘^Ϯ^’ after the PRM value and purple shading indicates a significant difference (logistic regression *p*<0.01) of metric values between PRM and individuals that survived capture.
(PDF)

S4 Table Blood parameters from each species of shark sampled, depending on at vessel condition or post-release fate. PRM = Post-release mortality; AVM = at-vessel mortality. Values are presented as mean ± SD. The numbers of animals in each category (*N*) are included, but note that this sample size is not necessarily the same for all parameters reported, as certain parameters were not necessarily available for all individuals, particularly for AVM and Alive at vessel individuals that were not tracked post-release. Blood pH values are temperature corrected according to methods reported in the text. An ‘*’ after the AVM value and blue shading indicates a significant difference (logistic regression *p*<0.01) of metric values between AVM and Alive at vessel individuals, and a ‘^Ϯ^’ after the PRM value and purple shading indicates a significant difference (logistic regression *p*<0.01) of metric values between PRM and individuals that survived capture.
(PDF)
