# Supplementary material for: Connecting post-release mortality to the physiological stress response of large coastal sharks in a commercial longline fishery
Source: PLoS One. 2021 Sep 15;16(9):e0255673. doi: 10.1371/journal.pone.0255673 (PMC8443047; doi:10.1371/journal.pone.0255673)
Supplement: S1 Table — Guidelines were first set by Hueter et al., (2006) [53]. (PDF) [file pone.0255673.s001.pdf]

S1 Table. Description of how condition index scores were assigned for released sharks.

Condition Index Score	Behavioral characterization
1: Good	Strong tailbeats, fast swimming and equilibrium upheld immediately upon release
2: Fair	Slow but consistent swimming immediately upon release, equilibrium upheld
3: Poor	Erratic or atypical swimming upon release, equilibrium mostly upheld
4: Very poor	Alive upon release, but weak or no swimming observed, inability to uphold equilibrium
5: Dead	Dead prior to release

Guidelines were first set by Hueter et al., (2006).
