# Supplementary material for: Connecting post-release mortality to the physiological stress response of large coastal sharks in a commercial longline fishery
Source: PLoS One. 2021 Sep 15;16(9):e0255673. doi: 10.1371/journal.pone.0255673 (PMC8443047; doi:10.1371/journal.pone.0255673)
Supplement: S2 Table — Metrics are divided by categories, which are also referred to in the text. (PDF) [file pone.0255673.s002.pdf]

S2 Table. All at-vessel metrics that were used in analyses.

Category	Metric
Behavioural metrics	
	Release condition
	Nictitating membrane reflex
	Bite reflex
	Flex reflex
	Equilibrium reflex
Environmental metrics	
	Water temperature
	Dissolved oxygen level
Morphological metrics/	
at-vessel measurements	Time on line
	Total length
	Girth
	Sex
Blood metrics	
Blood composition	pH
	Hematocrit
Metabolites	Lactate
	Glucose
Electrolytes	K^+
	Na^+
	Mg^{2+}
	Ca^{2+}
	Cl^-

Metrics are divided by categories, which are also referred to in the text.
