# Supplementary material for: Connecting post-release mortality to the physiological stress response of large coastal sharks in a commercial longline fishery
Source: PLoS One. 2021 Sep 15;16(9):e0255673. doi: 10.1371/journal.pone.0255673 (PMC8443047; doi:10.1371/journal.pone.0255673)
Supplement: S3 Table — PRM = Post-release mortality; AVM = at-vessel mortality. Values are presented as mean ± SD. Reflexes (nictitating membrane (NM), flex, bite, and equilibrium) were scored as a ‘1’ if unimpaired and a ‘0’ if impaired or absent, and release condition was scored as 1 to 4 based on swimming ability, with 1 indicating strong ability and 4 poor ability. The numbers of animals in each category (N) are included, but note that this sample size is not necessarily the same for all parameters reported, as certain parameters were not necessarily available for all individuals, particularly for AVM and Alive at vessel individuals that were not tracked post-release. An ‘*’ after the AVM value and blue shading indicates a significant difference (logistic regression p<0.01) of metric values between AVM and Alive at vessel individuals, and a ‘Ϯ’ after the PRM value and purple shading indicates a significant difference (logistic regression p<0.01) of metric values between PRM and individuals that survived capture. (PDF) [file pone.0255673.s003.pdf]

S3 Table. At-vessel measurements from each species of shark sampled depending on at vessel condition or post-release fate.

Species	Category	*N*	TOL	TL	Release condition	NM reflex	Bite reflex	Flex reflex	Equilibrium reflex	Water temp. (°C)	Dissolved oxygen
Sandbar	AVM	1	723	190	NA	NA	NA	NA	NA	19.8	7.3
	Alive at vessel	184	241 ± 225	194 ± 17	2.3 ± 0.7	0.88 ± 0.33	0.79 ± 0.41	0.88 ± 0.33	0.99 ± 0.09	21.9 ± 2.5	7.3 ± 0.7
	PRM	4	568 ± 380	206 ± 9	3.3† ± 0.5	0.33† ± 0.58	1 ± 0	0.67 ± 0.58	1 ± 0	21.7 ± 1.6	7.2 ± 0.3
	Survived	126	270 ± 225	201 ± 11	2.2 ± 0.7	0.89 ± 0.32	0.77 ± 0.42	0.88 ± 0.33	0.99 ± 0.10	21.5 ± 2.6	7.3 ± 0.8
Blacktip	AVM	108	234* ± 225	141 ± 20	NA	NA	NA	NA	NA	27.3 ± 3.8	6.5 ± 0.7
	Alive at vessel	184	115 ± 135	148 ± 19	2.3 ± 0.9	0.20 ± 0.40	0.36 ± 0.48	0.62 ± 0.49	0.89 ± 0.32	26.9 ± 3.1	6.5 ± 0.6
	PRM	44	117 ± 138	152 ± 15	2.7† ± 0.9	0.14 ± 0.35	0.36 ± 0.48	0.64 ± 0.48	0.79† ± 0.42	28.9 ± 2.8	6.1 ± 0.6
	Survived	61	135 ± 179	158 ± 15	2.0 ± 0.8	0.24 ± 0.43	0.36 ± 0.48	0.62 ±0.50	0.98 ± 0.13	27.5 ± 3.3	6.4 ± 0.8
Tiger	Alive at vessel	126	233 ± 249	202 ± 59	2.4 ± 0.8	0.93 ± 0.25	0.96 ± 0.21	0.81 ± 0.39	1 ± 0	23.4 ± 4.5	7.1 ± 0.94
	PRM	1	234	199	1	1	0	1	1	18.8	8.1
	Survived	51	312 ± 280	197 ± 34	2.3 ± 0.8	0.96 ±0.21	0.63 ± 0.49	0.78 ± 0.42	1 ± 0	22.4 ± 5.2	7.3 ± 1.1
Spinner	AVM	34	701* ± 174	175 ± 33	NA	NA	NA	NA	NA	18.8 ± 3.4	8.1 ± 0.85
	Alive at vessel	21	385 ± 254	185 ± 20	3.0 ± 0.9	0.40 ± 0.51	0.93 ± 0.26	0.87 ± 0.35	0.73 ± 0.46	19.7 ± 3.3	7.5 ± 1.1
	PRM	10	422 ± 248	192 ± 11	3.6† ± 0.7	0.33 ± 0.50	1 ± 0	0.89 ± 0.33	0.56 ± 0.53	19.7 ± 4.2	7.6 ± 1.3
	Survived	4	423 ± 313	174 ± 25	2.3 ± 0.5	0.50 ± 0.71	0.5 ± 0.71	1 ± 0	1 ± 0	19.0 ± 2.1	7.6 ± 1.3
Bull	Alive at vessel	36	167 ± 226	202 ± 45	2.4 ± 0.9	0.25 ± 0.46	0.63 ± 0.52	1 ± 0	1 ± 0	25.3 ± 3.1	6.8 ± 0.7
	PRM	1	103	265	3	1	0	1	1	31.0	5.1
	Survived	13	257 ± 315	218 ± 24	2.4 ± 0.9	0.14 ± 0.38	0.71 ± 0.49	1 ± 0	1 ± 0	26.6 ± 3.9	6.5 ± 0.7
Blacknose	AVM	56	212* ± 194	104 ± 13	NA	NA	NA	NA	NA	26.0 ± 4.3	6.8 ± 0.8
	Alive at vessel	178	109 ± 91	104 ± 13	2.4 ± 1.0	0.89 ± 0.32	0.63 ± 0.50	1 ± 0	0.95 ± 0.23	25.8 ± 2.4	6.7 ± 0.4
	PRM	1	157	111	3	1	1	1	1	23.6	7.2

PRM = Post-release mortality; AVM= at-vessel mortality. Values are presented as mean ± SD. Reflexes (nictitating membrane (NM), flex, bite, and equilibrium) were scored as a '1' if unimpaired and a '0' if impaired or absent, and release condition was scored as 1 to 4 based on swimming ability, with 1 indicating strong ability and 4 poor ability. The numbers of animals in each category (*N*) are included, but note that this sample size is not necessarily the same for all parameters reported, as certain parameters were not necessarily available for all individuals, particularly for AVM and Alive at vessel individuals that were not tracked post-release. An '*' after the AVM value and blue shading indicates a significant difference (logistic regression $p<0.01$) of metric values between AVM and Alive at vessel individuals, and a '†' after the PRM value and purple shading indicates a significant difference (logistic regression $p<0.01$) of metric values between PRM and individuals that survived capture.
