# Supplementary material for: Connecting post-release mortality to the physiological stress response of large coastal sharks in a commercial longline fishery
Source: PLoS One. 2021 Sep 15;16(9):e0255673. doi: 10.1371/journal.pone.0255673 (PMC8443047; doi:10.1371/journal.pone.0255673)
Supplement: S4 Table — PRM = Post-release mortality; AVM = at-vessel mortality. Values are presented as mean ± SD. The numbers of animals in each category (N) are included, but note that this sample size is not necessarily the same for all parameters reported, as certain parameters were not necessarily available for all individuals, particularly for AVM and Alive at vessel individuals that were not tracked post-release. Blood pH values are temperature corrected according to methods reported in the text. An ‘*’ after the AVM value and blue shading indicates a significant difference (logistic regression p<0.01) of metric values between AVM and Alive at vessel individuals, and a ‘Ϯ’ after the PRM value and purple shading indicates a significant difference (logistic regression p<0.01) of metric values between PRM and individuals that survived capture. (PDF) [file pone.0255673.s004.pdf]

S4 Table. Blood parameters from each species of shark sampled depending on at vessel condition and post-release outcome.

Species	Category	*N*	pH	Hematocrit (%)	Lactate (mmol L^{-1})	Glucose (mmol L^{-1})	K^+ (mmol L^{-1})	Na^+ (mmol L^{-1})	Cl^- (mmol L^{-1})	Mg^{2+} (mmol L^{-1})	Ca^{2+} (mmol L^{-1})
Sandbar	AVM	1	7.1	NA	38.1*	120.0	9.6*	291.9	262.8*	1.4*	2.6
	Alive at vessel	184	7.4 ± 0.1	25.7 ± 2.9	9.2 ± 7.9	30.5 ± 43.3	5.3 ± 0.6	283 ± 9	284 ± 7	1.0 ± 0.1	2.6 ± 0.1
	PRM	4	7.1† ± 0.2	27.6 ± 2.8	26.6† ± 5.8	64.5 ± 75.7	6.7† ± 1.3	287 ± 4	274† ± 11	1.1† ± 0.2	2.7 ± 0.1
	Survived	126	7.4 ± 0.1	25.6 ± 2.8	8.7 ± 7.4	29.3 ± 41.9	5.3 ± 0.5	283 ± 9	284 ± 7	1.0 ± 0.1	2.6 ± 0.1
Blacktip	AVM	108	6.6* ± 0.1	23.9 ± 8.2	35.7* ± 9.4	79.3 ± 34.6	10.9* ± 3.4	293 ± 11	276* ± 35	1.4* ± 0.4	3.2* ± 0.2
	Alive at vessel	184	7.2 ± 0.2	26.3 ± 4.1	23.4 ± 13.8	90.5 ± 46.2	6.4 ± 1.2	297 ± 15	288 ± 11	1.2 ± 0.3	3.0 ± 0.2
	PRM	44	7.1† ± 0.2	25.9 ± 4.4	29.8† ± 13.3	99.4 ± 42.9	6.9† ± 1.4	299 ± 15	285 ± 12	1.3† ± 0.2	3.0 ± 0.2
	Survived	61	7.3 ± 0.2	26.7 ± 4.2	17.4 ± 11.7	86.1 ± 47.4	5.9 ± 0.7	295 ± 15	290 ± 8	1.2 ± 0.2	3.0 ± 0.1
Tiger	Alive at vessel	126	7.5 ± 0.2	25.9 ± 4.8	4.7 ± 5.2	108.7 ± 53.1	5.3 ± 0.6	278 ± 10	283 ± 8	1.0 ± 0.2	2.8 ± 0.1
	PRM	1	7.6	28.2	1.5	123	5.0	258.9	264.3	0.9	2.6
	Survived	51	7.5 ± 0.1	26.6 ± 4.7	4.4 ± 5.1	115.5 ± 43.6	5.2 ± 0.6	276 ± 9	281 ± 8	1.0 ± 0.1	2.7 ± 0.1
Spinner	AVM	34	6.8* ± 0.2	32.6 ± 11.3	40.6* ± 12.5	90.4 ± 48.1	12.5* ± 4.2	283* ± 9	267* ± 10	1.4* ± 0.2	2.9 ± 0.2
	Alive at vessel	21	7.3 ± 0.2	32.2 ± 3.4	23.6 ± 13.8	80.0 ± 67.7	6.4 ± 1.3	299 ± 13	284 ± 12	1.2 ± 0.1	2.9 ± 0.2
	PRM	10	7.3 ± 0.2	32.6 ± 3.9	30.6 ± 13.8	93.5 ± 71.9	6.9 ± 1.5	301 ± 16	282 ± 16	1.2 ± 0.1	2.9 ± 0.2
	Survived	4	7.4 ± 0.1	30.6 ± 3.3	13.5 ± 9.8	99.2 ± 65.0	6.2 ± 0.9	296 ± 12	286 ± 7	1.2 ± 0.2	2.8 ± 0.1
Bull	Alive at vessel	36	7.2† ± 0.2	24.3 ± 3.5	9.6 ± 6.8	79.1 ± 37.7	6.0 ± 0.6	295 ± 10	296 ± 8	1.2 ± 0.2	3.0 ± 0.2
	PRM	1	6.7	18.9	20.4	102	7.2	286.8	291.9	1.4	3.2
	Survived	13	7.3 ± 0.2	24.8 ± 3.2	8.8 ± 6.3	77.3 ± 38.7	5.9 ± 0.5	296 ± 10	297 ± 8	1.2 ± 0.2	2.9 ± 0.2
Blacknose	AVM	56	6.6* ± 0.1	23.0 ± 8.1	34.4* ± 14.2	107.4 ± 23.6	12.4* ± 5.5	292 ± 8	276* ± 12	1.5 ± 0.2	3.0* ± 0.1
	Alive at vessel	178	7.2 ± 0.2	26.7 ± 4.7	18.5 ± 12.8	107.5 ± 51.3	6.2 ± 0.9	296 ± 9	287 ± 8	1.3 ± 0.3	2.9 ± 0.2
	PRM	1	6.9	30.7	17.4	78	5.9	305	287	1.2	2.8

PRM = Post-release mortality; AVM= at-vessel mortality. Values are presented as mean ± SD. The numbers of animals in each category (*N*) are included, but note that this sample size is not necessarily the same for all parameters reported, as certain parameters were not necessarily available for all individuals, particularly for AVM and Alive at vessel individuals that were not tracked post-release. Blood pH values are temperature corrected according to methods reported in the text. An '*' after the AVM value and blue shading indicates a significant difference (logistic regression $p<0.01$) of metric values between AVM and Alive at vessel individuals, and a '†' after the PRM value and purple shading indicates a significant difference (logistic regression $p<0.01$) of metric values between PRM and individuals that survived capture.
